# The National Institutes of Health measure of Healing Experience of All Life Stressors (NIH-HEALS): Factor analysis and validation

**DOI:** 10.1371/journal.pone.0207820

**Published:** 2018-12-12

**Authors:** Rezvan Ameli, Ninet Sinaii, María José Luna, Julia Cheringal, Brunilde Gril, Ann Berger

**Affiliations:** 1 National Institute of Mental Health, National Institutes of Health, Bethesda, Maryland, United States of America; 2 Clinical Center, National Institutes of Health, Bethesda, Maryland, United States of America; 3 Northwestern University, Chicago, Illinois, United States of America; 4 Walter Reed National Military Medical Center, Bethesda, MD, United States of America; 5 National Cancer Institute, National Institutes of Health, Bethesda, MD, United States of America; University of Twente, NETHERLANDS

## Abstract

Two hundred patients with severe and/or life-threatening disease were recruited form the NIH Clinical Center and participated in the validation of the NIH-HEALS, which included exploratory factor analysis, principal component analysis, reliability, convergent validity, and divergent validity analyses. Item-reducing principal components analysis and internal consistency and split-half reliability demonstrated excellent internal consistency and split-half reliability (Cronbach’s alpha = 0.89, split-half reliability = 0.95). Exploratory factor analysis revealed a three-factor structure, namely Connection (including religious, spiritual, and interpersonal), Reflection & Introspection, and Trust & Acceptance. Seven items were not retained. Convergent and divergent validity of 35-item NIH-HEALS against other validated measures of healing and spirituality provided strong evidence for its validity. As predicted, the Healed factor of the Self-Integration Scale (SIS), and Meaning, Peace, and Faith factors of the Functional Assessment of Chronic Illness Therapy-Spiritual Well-Being-12 Scale (FACIT-SP12) were all positively and significantly correlated with the NIH-HEALS and its three factors. Divergent validity was also confirmed by the significant negative correlation between the NIH-HEALS and the Codependent factor on the SIS. Confirmatory Factor Analyses revealed good model fit by GFI (0.96), adjusted GFI (0.95), SRMR (0.077), and RMSEA (0.065), supporting the use of the NIH-HEALS with 35 items.

## Introduction

During the course of diagnosis or treatment, some patients with a severe and/or life-threating illness can experience positive psychological, social, and spiritual change irrespective of their disease outcome [1–6]. Phenomena related to psych-social-spiritual healing have been reported in analyses of psychosocial adjustment trajectories among breast cancer patients [7], patients diagnosed with head and neck cancer[8] and in trauma victims [6]. Psycho-social-spiritual healing has been identified and often described in the literature by palliative care researchers and clinicians [9–13]. Kearney [12] identifies healing as an inner agency that gives a degree of relief from suffering, an inner agency that the patient must find within the depths of his or her own psyche. The concept of “total pain” [9] refers to a complex of physical, emotional, social, and spiritual factors that contribute to suffering. Egnew [14], identifies “the personal experience of the transcendence of suffering” (p258) to be the essence of healing, i.e. healing brings about a measure of relief from suffering. Benor emphasizes the holistic nature of healing experience as involving body, emotions, mind, relationships, and spirit [15]. Recent studies [16–18] have illustrated potential mechanisms of psychosocial spiritual healing. Reduction of suffering [12], restoring well-being [19–21], and achieving personal growth above and beyond the pre-disease state are considered to be important angles when considering the concept of healing. Meza and Fahome [22] define healing as the human experience of self-discovery and transformation that results in a sense of being whole and connected. They used the theoretical model suggested by Miller et al [23] and developed a scale, Self-Integration Scale (SIS), to assess the psychological and social construct of healing.

The conceptual underpinning of our work came from palliative care providers who observed psychological, social, and spiritual well-being in some patients during the adversities of serious terminal illnesses even when it led to death. Our experience with literature review and qualitative interviews [24] pointed to the multifaceted experience of healing similar to Benor’s formulation [15] and guided the original compilation of questionnaire items to assess the healing experience. We believe the assessment of the degree of psycho-social-spiritual healing in the face of stressful or even life-threatening circumstances can provide important information for patient specific interventions aimed at enhanced coping, adjustment, well-being, quality of life, and promoting a state of healing. In particular, assessment of healing in palliative medicine, which is concerned with the relief of physical, psychological, social, and spiritual pain and suffering, is of paramount importance. The literature is clear about the importance of meaning as a central component to healing [20, 25–28], however, it does not provide a set of factors that contribute to healing and the healing experience with specificity [18].

We have previously reported three studies [18, 29, 30] that were conducted at the National Institutes of Health (NIH), Clinical Center (CC), Pain and Palliative Care Service (PPCS) that elucidate the process of developing the HEALS, the predecessor to the NIH-HEALS, (see reference #18 for detailed description of the multi-step process and the pilot study) as a measure of the construct of healing. In the pilot study, factor analysis was conducted with the HEALS 48-item version. Following the pilot factor analysis, cognitive interviewing [31] was conducted to improve the items and item selection and to ensure that the items were linguistically and culturally sensitive. Cognitive interviewing resulted in the reduction of items from 48 to 42. The present study describes the processes recommended for a newly developed measure [32, 33] and describes the NIH-HEALS exploratory factor analysis, principal component analysis, reliability, convergent and divergent validity, and confirmatory factor analysis.

## Methods

### Subjects & procedure

The NIH Office of Human Subject Research Protection (OHSRP) approved the study. The study was performed in a de-identified and anonymized manner, i.e. did not include patient names or medical record numbers, thus, written consent was waved by the OHSRP. The packet of questionnaires included an informed consent sheet that described the study, the purpose of the study, the voluntary nature of the participation, the nature of the information requested, the approximate time it takes to complete the questionnaires, and an explicit explanation that declining to participate did not affect the patient’s care at the NIH Clinical Center (S1 File). The Consent Information also included contact information for one of the investigators (RA) in case of additional questions or concerns. If the patient verbally consented to participate, then a numbered packet of self-report questionnaires was given to the participant. PPCS staff and two Special Volunteers received in-service from PPCS research staff (AB, RA, MJL, JHC) regarding the project. A PPCS representative, i.e. research associates (MJL, JHC), PPCS clinicians, or one of the two Special Volunteers, approached the patients in several Clinical Center hospital units and Outpatient clinics while the patients were in their hospital rooms or waiting for their medical appointments in outpatient clinics. The PPCS representative first verbally described the study and if the patient expressed interest, they were presented with the written Consent Information and the packet of questionnaires. PPCS representatives were available while patients completed the questionnaires. All patients were already involved in experimental treatments and research projects for the study and treatment of their particular disease at the NIH Clinical Center. They were in various stages of their treatment and recovery. Some patients were in remission in response to the investigative treatments. Two hundred consecutive NIH Clinical Center patients involved in experimental clinical research who consented to participate, were included in this current study. The recruitment took place from June to December of 2017.The eligibility criteria for this study included age of 18 or above, the ability to read and write in English, and the presence or history of a serious and/or life-threatening disease. These included but were not limited to various forms of advanced/metastatic cancer in one or several organs (e.g. lung, liver, pancreas, thyroid, thymus, ovaries, prostate, colon, stomach, kidney, blood, brain, and skin), blood dyscrasias (e.g. sickle cell anemia, aplastic anemia), graft vs. host disease (GVHD), as well as severe and rare genetic conditions (e.g. Camurati-Englemann Disease, Carney Complex Disease, Familial Dysautonomia, Job’s Syndrome, von Hippel-Lindau Disease, Myelodysplastic Syndrome, Neuromyelitis Optic or Devic’s Syndrome, Neurofibromatosis). Table 1 summarizes patient demographics by self-report including gender, age, race/ethnicity, marital status, religious affiliation, education, employment status, medical diagnosis, estimated duration of illness, the patient’s perception of their current severity of their illness, psychiatric comorbidity, perceived stress level, perceived level of social support, overall health status, and overall quality of life (S2 File).

**Table 1:** Subject demographics of enrolled participants with serious and life-threatening disease.

		*n = 200*	%*
Gender	Female	103	53
	Male	90	47
	Total	193	
Age, in years (mean ± SD, range)	50.2 ±15.5, 18–89	184	
Race	American Indian/Alaska Native	1	1
	Asian	13	7
	Black or African American	30	16
	Caucasian	139	72
	Mixed/Two or More	5	3
	Other	4	2
	Total	192	
Ethnicity	Hispanic or Latinx	13	7
	Not Hispanic or Latinx	176	93
	Total	189	
Marital Status	Single	42	22
	Married	116	60
	Divorced/Separated	21	11
	Widowed	7	4
	Living with Partner	3	2
	Other	3	2
	Total	192	
Religious Affiliation	Christianity	126	66
	Islam	3	2
	Hinduism	2	1
	Buddhism	2	1
	Judaism	5	3
	Agnostic	10	5
	Atheist	11	6
	Not Affiliated	23	12
	Other	8	4
	Total	190	
Education	Grade School	1	1
	High School/GED	25	13
	Vocational Training	6	3
	Some College/University	43	22
	Completed College/University	65	34
	Graduate School/Advanced Degree	52	27
	Other	1	1
	Total	193	
Employment Status	Full Time	60	31
	Part Time	17	9
	Not Employed	61	32
	Retired, Disabled, or Other	54	28
	Total	192	
Medical Diagnosis	Cancer (advanced/metastatic)	128	70
	Severe and/or Rare Non-Genetic conditions	20	11
	Blood Dyscrasias	17	9
	Severe and/or Rare Genetic conditions	16	9
	AIDS	1	1
	Total	182	
Estimated Duration of Illness, in months (years) (mean ± SD, median, range)	99 (~8 yrs.) ±121 (~10 yrs.), 48 (~4 yrs.) 1–600 (~50 yrs.)	176	
Severity of Illness	Extremely severe	80	43
	Severe	67	36
	Moderate	27	14
	Mild	10	5
	Not Severe	3	2
	Total	187	
Psychiatric Comorbidity (Depression, Anxiety, Bipolar, etc.)	35 of 192 Participants		18
Perceived Stress Level	Extreme	6	3
	Severe	20	11
	Moderate	88	47
	Mild	55	29
	No Stress	18	10
	Total	187	
Perceived Level of Social Support	Excellent Support	107	57
	Good Support	54	29
	Some Support	26	14
	No Support	0	0
	Total	187	
Overall Health Status	Excellent	4	2
	Good	37	20
	Satisfactory/fair	45	24
	Manageable	77	41
	Poor	26	14
	Total	189	
Overall Quality of Life	Excellent	28	15
	Good	69	37
	Satisfactory/fair	40	21
	Manageable	36	19
	Poor	15	8
	Total	188	

*Data are frequencies and rounded percentages unless otherwise specified. SD = standard deviation. Percentages may not total 100% due to rounding.

## Instruments

All participants received a package of questionnaires that included the HEALS, the Functional Assessment of Chronic Illness Therapy-Spiritual Well-Being (FACIT-Sp-12)[34]) the Self Integration Scale (SIS) [22]) version 2.1. The latter two measures were administered to assess convergent and divergent validity. In addition, we were interested in exploring the relationship between the NIH-HEALS (S3 File), and mindfulness, resilience, and trauma history and therefore the Mindful Attention Awareness Scale (MAAS) [35], The Connor-Davidson Resilience Scale (CD-RISC 10 item version) [36], and Life Events Checklist 5 (LEC-5) [37] were also administered and will be reported separately.

**HEALS** is a 42-item self-report questionnaire developed by the NIH Clinical Center PPCS as a comprehensive measure of psychological, social, religious and spiritual healing experience that assesses an individual’s psycho-social-spiritual mechanisms for coping during life’s difficult situations and/or life-threatening–challenges (S4 File) [18]. It is scored on a five-point Likert scale from Strongly Disagree (1) to Strongly Agree (5). Five items require reversed scoring. Previously, a pilot study was conducted to explore the factor structure and to also remove or revise individual items. In the pilot study HEALS demonstrated very good internal consistency (Cronbach’s *a =* 0.94) and a four factor structure including religion (Cronbach’s *a =* 0.98) example item: My personal religious practice is important to me), spirituality (Cronbach’s *a* = 0.88) example item: I have a heightened sense of gratitude), intrapersonal relationships (Cronbach’s *a* = 0.84) example item: I am content with my life), and interpersonal relationships (Cronbach’s *a* = 0.83) example item: connection with my family has become my highest priority). This pilot study was the first step towards a better understanding of the HEALS items and factor structure. As was planned at the time, a larger study was needed to further characterize the HEALS. The current study was therefore designed to re-examine the factorial structure, further evaluate individual items, examine reliability, assess convergent and divergent validity, and conduct confirmatory factor analysis with a new sample.

**FACIT-Sp12** is a 12-item self-report questionnaire that measures spiritual well-being in people with cancer and other chronic illnesses and is scored on a five-point Likert scale from Not at All (0) to Very Much (5) (S5 File) [34, 38]. It is a part of the larger FACIT measurement system (http://www.FACIT.org). FACIT-Sp12 is one of the most widely used instruments in research of spiritual well-being and is based on a broad definition of spirituality. It has high internal consistency (Cronbach’s *a* = 0.87). Some reports recommend a two factor solution, i.e. peace and meaning [39]. A three-factor structure has been reported in cancer survivors [34, 38]. The factors include meaning (example item: I have a reason for living), peace (example item: I feel peaceful), and faith (example item: I find comfort in my faith). In the current study, we utilized the three factor structure for our analysis.

**SIS version 2.1** [22] is an 18-item self-report instrument that incorporates a five-point Likert scale from Very Rarely (1) to Most of the Time (5) (S6 File). SIS is shown to be a valid and reliable scale for assessing the psychological and social aspects of the healing construct. It has high internal consistency (Cronbach’s *a* = 0.86). The factorial structure of this instrument points to two factors: healed (example item: When my future is uncertain, I have a basic sense of trust that things will turn out OK) and codependent (example item: I feel that others control my life), where one factor (healed) converges with the concept of healing and the other (codependent) does not. The items of the co-dependent factor which is derived from the Spann-Fischer Codependency Scale [40] were intentionally chosen as a diverging construct to healing and provided an excellent choice for assessing the NIH-HEALS divergent validity as well.

### Statistical analyses

Data were analyzed using SAS v9.4 (SAS Institute, Inc., Cary, NC). As applicable, data are described using frequencies and percentages or means and standard deviations. For purposes of data reduction and re-examination, PROC FACTOR in SAS was used to conduct Exploratory Factor Analysis (EFA) of Principal Components Analysis (PCA) utilizing an orthogonal rotation and varimax procedure. A factor loading threshold of >0.40 was used for retention. The modified and final NIH-HEALS measure included 35 retained items, of which, items 6, 23, 28, and 34 were reversed for scores. Correlation analysis using Spearman’s correlation coefficient was used for convergent and divergent validity, with coefficients >0.3 indicating moderate correlation and those >0.5 indicating strong correlation. Internal consistency was measured by Cronbach’s alpha [41] and split-half reliability [42] using the Spearman-Brown formula, with modest reliability thresholds generally at 0.70. Kaiser’s measure of sampling adequacy (MSA) was also computed, compared to levels of 0.8–0.9 that are considered good [43].

Confirmatory Factor Analysis (CFA) used the PROC CALIS procedure in SAS to verify the factor structure of the 35-item NIH-HEALS measure and allowed for the testing of the hypothesis to confirm the presence of a relationship between the observed variables and their underlying latent constructs. These Structural Equation Modeling (SEM) techniques rely on several statistical tests to determine the adequacy of model fit to the data [44, 45]. Several indices of model fit were used. The chi-square test indicates the amount of difference between expected and observed covariance matrices, where 0 indicates little differences between them, and a corresponding p-value greater than 0.05 would be desired for good model fit. Comparative Fit Index (CFI) is the discrepancy function adjusted for sample size, and levels greater than the range of 0.90–0.95 are considered acceptable and those >0.97 considered good for model fit [46]. Root Mean Square Error of Approximation (RMSEA) is related to the residual in the model, with acceptable values <0.08, and good model fit at <0.05 [47]. The Standardized Root Mean Square Residual (SRMR) has similar criteria for model fit. Other indices included the Non-Normed Fit Index (NNFI), with acceptable model fit at 0.95 and good at 0.97, and the Normed Fit Index (NFI) and Goodness-of-Fit Index (GFI), with acceptable fit at 0.90 and good at 0.95. Adjusted GFI suggests acceptable model fit at 0.85 and good fit at 0.90. Since data were not approximately normally distributed, the CFA model applied a Robust Maximum Likelihood (MLSB) estimation method and corrected chi-square. In addition, CFA utilized the mean and covariance structures for analysis. P-values <0.05 were considered statistically significant.

## Results

Two hundred patients participated in the study. Of the 193 participants who indicated their gender, 53% were female. The age ranged from 18–89 years with a mean of 50.2 (±15.5) years. The racial composition of the group included Caucasian (72%), Black or African American (16%), and Asian (7%). Seven percent of subjects reported Hispanic/Latinx ethnicity. The majority of participants were married (60%), Christian (66%), and educated (34% completed college/university and 27% completed graduate school/advanced degrees). In terms of employment, some were working full (31%) or part (9%) time (Table 1). Simple descriptive statistics for total and factor scores for the various scales used in the study are described in Table 2.

**Table 2:** Descriptive statistics of scores for the three-factor, 35-item NIH-HEALS measure, SIS, and FACIT-SP12 scale in study participants with serious and life-threatening disease.

Scale	Factors	n	Mean ± SD	Median (IQR)	Range	Range of Possible Scores
**NIH-HEALS**	Connection	196	37.7 ± 9.8	40 (31–46)	13–50	10–50
	Reflection & Introspection	192	54.8 ± 6.8	54 (50–60)	38–70	14–70
	Trust & Acceptance	194	40.4 ± 6.7	41 (36–45)	20–55	11–55
	Total	186	132.9 ± 18.6	134 (120–145)	87–172	35–175
**SIS**	Healed	195	34.7 ± 6.8	36 (29–40)	17–45	9–45
	Codependent	187	34.2 ± 6.9	35 (30–40)	14–45	9–45
	Total	185	68.7 ± 11.3	70 (61–78)	35–89	18–90
**FACIT-SP12**	Meaning	194	13.1 ± 3.2	14 (11–16)	3–16	0–16
	Peace	193	10.3 ± 3.7	11 (8–13)	0–16	0–16
	Faith	196	10.3 ± 5.2	12 (5–15)	0–16	0–16
	Total	192	33.7 ±10.0	35 (27–42)	6–48	0–48

All scores incorporate reverse response scoring, as applicable.SD = Standard Deviation; IQR = Inter-quartile range (25^th^-75^th^ percentile); NIH-HEALS = National Institutes of Health Healing Experiences of All Life Stressors; SIS = Self-Integration Scale version 2.1; FACIT-SP12 = Functional Assessment of Chronic Illness Therapy-Spiritual well-being -12.

**Fig 1:**
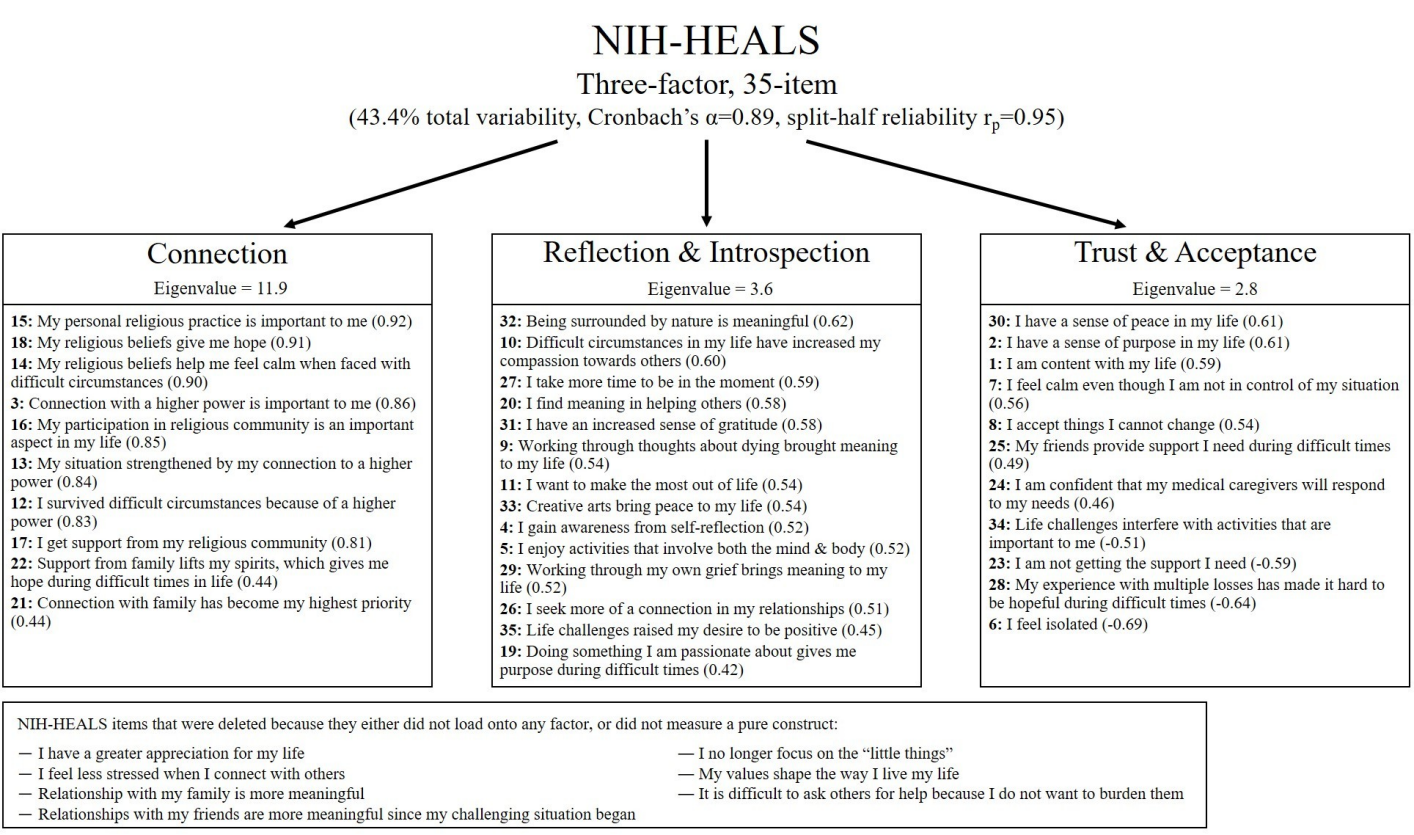
Three factor, 35-item NIH-HEALS measure. The three factors accounted for 43.4% of the total variability with excellent reliability (Cronbach’s alpha = 0.89, split-half reliability = 0.95). Retained items for each factor are listed along with their individual loadings (in parenthesis) from the exploratory factor analysis. Items 6, 23, 28, and 34 are reversed scored.

Exploratory factor analysis (EFA) yielded an initial ten factors with eigenvalues greater than the accepted 1.0, which explained 66.5% of the total variability. Cattell’s scree test indicated a break point between three and four factors, with eigenvalues ≥2.0 and 43.4% and 48.2% explained total variability, respectively. A three-factor NIH-HEALS measure generated the most pristine item loadings. A total of 7 items were excluded from the HEALS (see Fig 1 for a list of excluded items). There were two items that did not load on any factor (“It is difficult to ask others for help because I do not want to burden them” & “I no longer focus on the ‘little things’”), one that did not measure a pure construct (“I have a greater appreciation for my life”), and four items that did not meet the factor loading requirements (“I feel less stressed when I connect with others” & “My values shape the way I live my life” & “Relationships with my friends are more meaningful since my challenging situation began” & “Relationship with my family is more meaningful”). During this confirmatory phase, a minimum factor loading threshold of >0.40 was used for retention of items. Together, the item reduction and factor analyses led to the 35-item, three-factor NIH-HEALS measure (Fig 1). The eigenvalue for factor 1 (Connection) was 11.9, for factor 2 (Reflection & Introspection) was 3.6, and for factor 3 (Trust & Acceptance) was 2.8, all exceeding the accepted standard. The 35-item NIH-HEALS measure had persistently strong internal consistency (Cronbach’s alpha = 0.89) and split-half reliability (r_p_ = 0.95).

The NIH-HEALS total score and scores for each of its three factors were used for convergent and divergent validity determinations (Table 3). Convergent validity was measured against the SIS Healed factor, yielding strong positive correlations with the NIH-HEALS (r_s_ = 0.64, p<0.0001). Convergent validity of the NIH-HEALS was also tested against the FACIT-SP12 Meaning, Peace, and Faith factor scores (r_s_ = 0.62, p<0.0001; r_s_ = 0.60, p<0.0001; and r_s_ = 0.60, p<0.000, respectively). Divergent validity of the NIH-HEALS was measured against the SIS Codependent factor, yielding inverse correlations all around (r_s_ = -0.34, p<0.0001). These values supported criterion validity. Correlations between NIH-HEALS’ individual factors (Connect, Reflection & Introspection, and Trust & Acceptance) and SIS and FACIT-SP12 factors were all statistically significant and are reported in Table 3.

**Table 3:** Divergent and convergent validity results for the three-factor, 35-item NIH healing experience of All Life Stressors (NIH-HEALS) measure.

	NIH-HEALS Total Score	NIH-HEALS Factor 1 (Connection) Score	NIH-HEALS Factor 2 (Reflection & Introspection) Score	NIH-HEALS Factor 3 (Trust & Acceptance) Score
**Divergent Validity**				
SIS Codependent Factor Score	**-0.34** (p<0.0001)	**-0.16** (p = 0.0264)	**-0.15** (p = 0.0464)	**-0.58** (p<0.0001)
**Convergent Validity**				
SIS Healed Factor Score	**0.64** (p<0.0001)	**0.41** (p<0.0001)	**0.59** (p<0.0001)	**0.59** (p<0.0001)
FACIT-SP12 Meaning Score	**0.62** (p<0.0001)	**0.39** (p<0.0001)	**0.45** (p<0.0001)	**0.64** (p<0.0001)
FACIT-SP12 Peace Score	**0.60** (p<0.0001)	**0.35** (p<0.0001)	**0.51** (p<0.0001)	**0.72** (p<0.0001)
FACIT-SP12 Faith Score	**0.84** (p<0.0001)	**0.84** (p<0.0001)	**0.51** (p<0.0001)	**0.54** (p<0.0001)

Data are Spearman’s correlation coefficients and corresponding p-values.

SIS = Self-Integration Scale version 2.1; FACIT-SP12 = Functional Assessment of Chronic Illness Therapy-Spiritual well-being -12.

**Fig 2:**
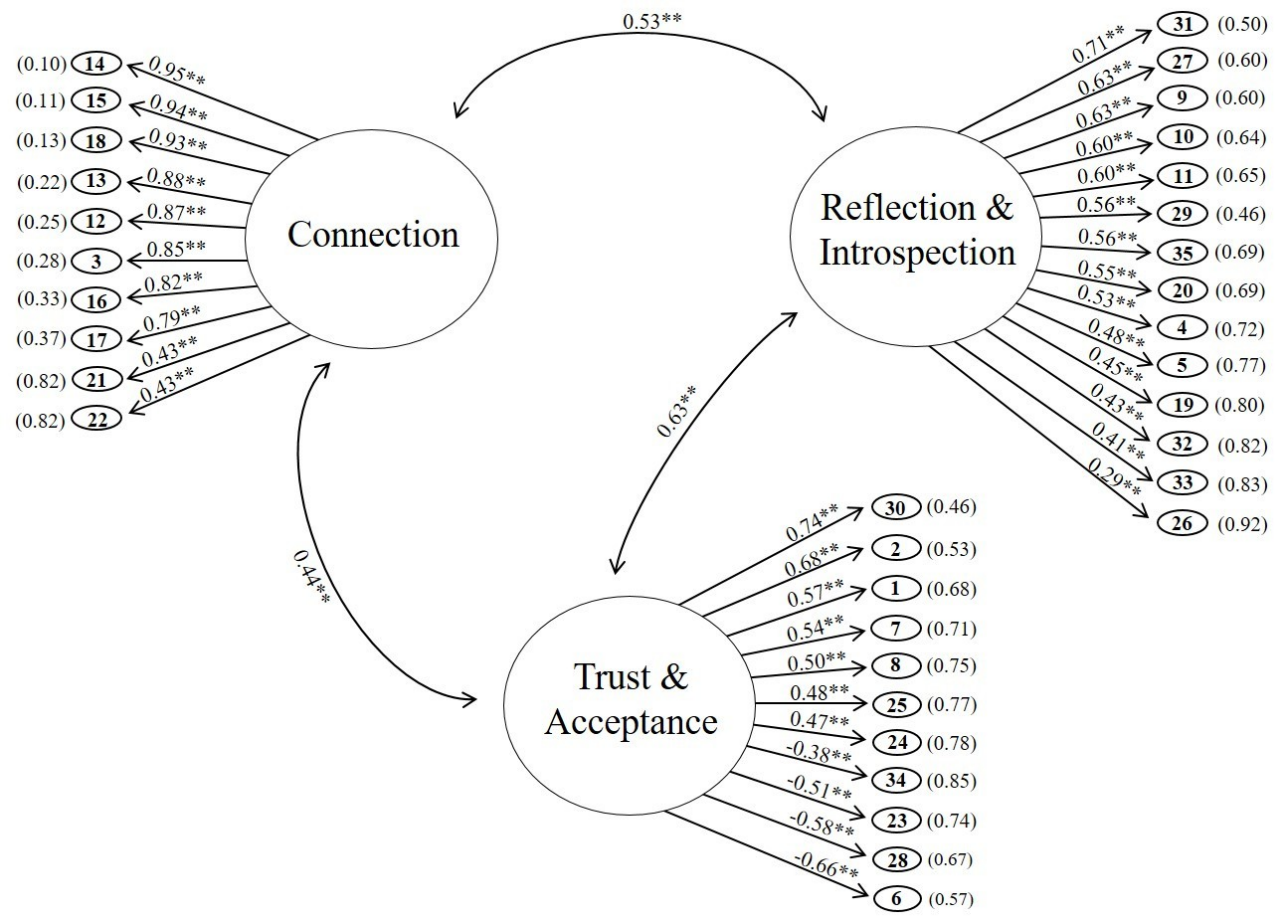
Path diagram of the three-factor, 35-item NIH-HEALS measure. Data are standardized coefficients from mean and covariance structure model of confirmatory factor analysis. The numbers in the ovals represent the NIH-HEALS items. Data in parenthesis following items are standardized error coefficients. **p<0.0001.

Confirmatory factor analysis assessed construct validity. Structural equation modeling (SEM) parameter estimates indicated that each of the NIH-HEALS items was significantly related to its corresponding factor. The standardized coefficients ranged from 0.43 to 0.95 for factor 1 (Connection), 0.29 to 0.71 for factor 2 (Reflection & Introspection), and -0.66 to 0.74 for factor 3 (Trust & Acceptance) (Fig 2). Several indices were used to assess goodness of fit for the three-factor, 35-item NIH-HEALS measure (Table 4). With the exception of the chi-square test (p<0.0001), model fit was shown to be good by GFI (0.96) and adjusted GFI (0.95), acceptable by SRMR (0.077) and RMSEA (0.065, 90% CL: 0.058–0.072), and reasonably close by Bentler CFI (0.86) and Bollen NNFI (0.86), overall supporting the three factor, 35-item NIH-HEALS measure as a good and appropriate model.

**Table 4:** Goodness-of-Fit indices for the three-factor, 35-item NIH healing experience of All Life Stressors (HEALS) measure.

Fit Index	Three-Factor, 35-Item HEALS	Standard Thresholds
Chi-Square/DF (p-value)	2.128/557 (p<0.0001)	Close to 0 (p > 0.05)
Bentler Comparative Fit Index (CFI)	0.8592	Acceptable: 0.90–0.95 Good: 0.97
Root Mean Square Error Approximation (RMSEA) (90% Confidence Limit)	0.0650 (0.0584, 0.0715)	Acceptable: <0.08 Good: <0.05
Standardized Root Mean Square Residual (SRMR)	0.0766	Acceptable <0.08 Good <0.05
Goodness-of-Fit Index (GFI)	0.9617	Acceptable: 0.90 Good: 0.95
Adjusted GFI (AGFI)	0.9543	Acceptable: 0.90 Good: 0.95
Bentler-Bonett Normed Fit Index (NFI)	0.7117	Acceptable: 0.90 Good: 0.95
Bollen Non-Normed Fit Index (NNFI)	0.8610	Acceptable: 0.95 Good: 0.97

## Discussion

The NIH-HEALS was developed to better understand and measure healing and factors that contribute to the experience of healing in the midst of significant life stressors including, but not limited to, severe and/or life-threatening disease. The assessment of healing and its various aspects can help identify areas of needed intervention to support patient-centered care and positive treatment outcomes. NIH-HEALS provides a comprehensive instrument for such as assessment. The steps to the development of the NIH-HEALS has included literature review, qualitative inquiry, expert review, considerations for existing measures to avoid redundancy [48], a pilot study to evaluate and review items to be included, and cognitive interviewing to refine item inclusion, and presently re-evaluation of the factor structure and refinement of item inclusion, the examination of convergent and divergent validly, and confirmatory factor analysis with a new sample of 200 patients from the NIH Clinical Center. Based on these studies, we now propose a 35-item, three-factor NIH-HEALS as a measure of psycho-social-spiritual healing. These factors include: 1. Connection—belief in and connection to a higher power, religion, religious community, and family; 2. Reflection & Introspection—finding meaning, purpose, gratitude and joy in nature, activities including those that connect mind and body, interconnectedness, present moment orientation, and an increased sense of awareness about the fragility of life; and 3. Trust & Acceptance—accepting what is, feeling resolved, feeling at peace, and trusting that caregivers, friends, and family will respond to needs as they arise.

As predicted, we found that the NIH-HEALS and its factors are significantly correlated with FACIT-Sp12 factors and the Healed factor of the SIS, providing evidence for its convergent validity. We further found evidence of the NIH-HEALS divergent validity and, as predicted, there was a negative correlation with the SIS Codependent factor. Construct validity was further examined by the use of confirmatory factor analysis providing evidence of the goodness of fit for the three-factor NIH-HEALS.

It is important to note that the NIH-HEALS three factors are not discrete constructs. Rather, they are related concepts that delineate psycho-social-spiritual elements that could contribute to the experience of healing.

In terms of the first factor, Connection, there is a great deal of information in the literature that support the importance of spiritual and/or religious practices, connection to a higher power, and organized religious activities in promotion of health and well-being during difficult times, including serious illness [34, 49–53]. Higher levels of religious involvement are associated with greater well-being, and mental and physical health [54–56]. The importance of religion and spiritual well-being in oncology and palliative care settings have been well documented [49]. Shaw and colleagues [57], in their review of 11 empirical studies examining the relationship between spirituality, religion, and posttraumatic growth, concluded that religion and spirituality can be beneficial in coping with the aftermath of trauma, which can lead to a deepening of religiousness or spirituality. Furthermore, the authors posit that positive religious coping, religious openness, readiness to face existential questions, religious participation, and intrinsic religiousness are related to posttraumatic growth. Pargament [58] proposed that positive religious coping can involve several aspects including benevolent reappraisal, spiritual support, surrender to a higher power, spiritual connection, and seeking religious direction that “uniquely equip individuals to respond to situations in which they come face-to-face with the limits of human power and control and are confronted with their vulnerability and finitude”. Religious coping has also been found to support and contribute to the integration of traumatic experiences [54, 56].

Social connections including family and friends are another important aspect of the NIH-HEALS Connection factor. Social connections undoubtedly impact the adjustment and quality of life when faced with adversity. Psychological and health benefits of social support and its connection to cardiovascular, neuroendocrine, and immune functions are well-documented [59–62]. In a well-known meta-analysis of 148 studies, including more than 308,000 men and women, Holt-Lundstad and colleagues [63] found that the boost in longevity in those with robust social ties is comparable to mortality difference between leading health indicators such as smoking, lack of exercise, and obesity. Those with poor social connections had on average 50% higher odds of death in the study's follow-up period (an average of 7.5 years) than people with more robust social ties. There is strong evidence that social isolation and loneliness significantly increase risks for premature mortality and the magnitude of the risks exceeds that of many leading health indicators [64]. The impact of social ties was found to be unrelated to gender, age, or health status [63].

The NIH-HEALS second factor, Reflection & Introspection, is consistent with finding meaning in activities, nature, art, and the importance of present moment orientation in the experience of healing. The question of meaning has become the subject of increasing theoretical and empirical interest [65] and the foundation for several psychological interventions in mental health, palliative care, and oncology settings [49]. Many of these interventions trace their origins to the work of Victor Frankl [66]. Experience of meaning in life and engagement in meaningful activities contribute to well-being and health [67–70]. Coherence, purpose, and significance are currently considered to be the three facets of meaning [71]. Martela and Steger [71] posit that in order to live as reflective beings and experience meaning in life, humans need to comprehend the world around them (coherence), find direction for their actions (purpose), and find worth in their lives (significance). In the context of adverse events, such as a cancer diagnosis, finding meaning in life appears as a salient coping mechanism to adjust to a life-threatening disease [72]. Diagnosis of severe and/or life- threatening disease, or other traumatic events for that matter, can shatter one’s sense of coherence, the predictability of events, a sense of basic safety, purpose, and significance. It follows that those who are able to restructure, comprehend, and accept their lives as it is, find joy and pleasure in activities, accept the changes, redefine their sense of purpose, and re-affirm their sense of being of significance in the world, are in a better position to experience healing. When the facets of meaning are examined closely, the NIH-HEALS factor 1, Connection, can also be considered under the umbrella of meaning-making. Both social and spirituality/religion connections contribute to the sense of meaning in life. In a systematic review investigating 22 studies, Moreno and Stanton [73] identified social relationships and spiritual/religious activities as key components to perceive life meaning and develop personal growth among patients with advanced cancer. George and Park [74] identified spirituality as a source of life meaning among patients with heart failure or cancer.

The NIH-HEALS third factor, Trust & Acceptance, taps into another rich area of inquiry. Accepting what is, integrating the new status of affairs, making sense of them coherently, demonstrating flexibility and resilience, and moving forward with a trusting attitude that one’s needs will be met by family, friends and caregivers, are other domains that contribute to a healing experience. In a trauma related study, it was found that basic trust significantly contributed to post traumatic growth [75]. Trzebinski and Zieba [76] differentiate between optimism, hope, and basic trust. The level of basic trust is considered a function of childhood experiences which is modified by later experiences in life. The orderliness of early home environment and predictability of early relationship with parents plant the seed for basic trust. A strong sense of basic trust allows one to accept trauma and loss, including a traumatic illness, with an initial but appropriate sadness and resignation and the ability to redirect attention to new and constructive coping strategies.

Another facet of Trust and Acceptance is highlighted by the studies focused on the importance of trust in caregivers, particularly in the context of severe and or life-threatening illness [77]. A review of the literature focusing on cancer patients and a qualitative interview study [78, 79] established that physician’s perceived competence, honesty, and patient-centered behaviors contribute to the enhancement of trust, which in turn, is related to the ease of communication, decrease in patient fear, and better treatment adherence.

In summary, the NIH-HEALS items were identified through non-theoretical qualitative patient interviews with individuals suffering from severe and/or life-threatening illness who demonstrated remarkably positive coping and attitude. These items tap into important areas of healing experiences, which include spirituality, religion, meaning in life, social ties, acceptance, and trust. We propose that the NIH-HEALS 35-item version provides a robust and statistically sound measure that captures important and diverse aspects of the healing experience.

There are limitations to the NIH-HEALS generalizability and usage. It is developed in a clinical research setting in the United States and therefore its usefulness in other settings and cultures would have to be reassessed and reestablished. In particular, the definition of healing and healing interventions can be quite diverse across cultures [80]. In addition, although the NIH-HEALS is a promising research tool, at this time, it would be inappropriate to use it for individual assessments or in clinical practice, since normative data for clinical populations and subgroups do not yet exist. Another limitation which impacts the generalizability of the study is that the education level in our sample was quite high with over 61% of the subjects reporting college level or higher education. Although this is common among patients who desire to participate in NIH research studies[81], the percentage of Americans aged 25 or older with at least a bachelor’s degree is 32.5% in the general population according to the census bureau [82]. Future investigations of various populations including clinically identified “healed” individuals, individuals with trauma exposure other than severe and/or life-threatening medical illness (such as war veterans), and control subjects without known serious medical or psychiatric conditions, will further refine the concept of healing and the characterization of the NIH-HEALS and its utility. In addition, further research with new samples can shed more light on the factor structure. Investigations aimed at the NIH-HEALS sensitivity to change with intervention (i.e. pre-post intervention) will also be of great interest. In addition, future research could consider focusing on minority subgroups to further investigate the mechanism(s) involved in psycho-social-spiritual healing in these subgroups.

## Supporting information

S1 File Consent information. (DOCX)

S2 File Demographic questionnaire. (DOCX)

S3 File NIH-HEALS. (DOCX)

S4 File HEALS 42 item. (DOCX)

S5 File FACIT-SP 12. (DOCX)

S6 File SIS version 2.1. (DOCX)

S1 Data set Minimal underlying data. (XLSX)
