# Supplementary material for: The National Institutes of Health measure of Healing Experience of All Life Stressors (NIH-HEALS): Factor analysis and validation
Source: PLoS One. 2018 Dec 12;13(12):e0207820. doi: 10.1371/journal.pone.0207820 (PMC6291293; doi:10.1371/journal.pone.0207820)
Supplement: S1 File — (DOCX) [file pone.0207820.s001.docx]

**Consent Information**

At the Pain and Palliative Care service (PPCS), we have developed a psycho-social spiritual measure, the Healing Experience in all Life Stressors (HEALS), that focuses on the healing experience after individuals have been diagnosed with a serious illness. We would like to further validate this tool so that in the future we can use it to identify individuals who may benefit from additional interventions to cope with their illness. Your participation will help us to find out if the HEALS is helpful to the work we do. We are also collecting this data for research purposes. We will ask you to complete 6 short questionnaires which usually take about 15-20 minutes to complete. The questions will be focused on basic information, health information, life events, spiritual and religious beliefs, and day-to-day experiences. Your participation is completely voluntary. Please know that declining to participate will not affect your care or your relationship with any of the clinical staff here at the Clinical Center. In addition, your responses will be collected anonymously.

You can call or email Rezvan Ameli, Ph.D. ([Rezvan.ameli@NIH.Gov](mailto:Rezvan.ameli@NIH.Gov), PPCS Tel: 301-594-9767) with any questions or concerns about this activity. If you prefer Dr. Ameli to contact you instead, please request this from the PPCS representative.
