# Supplementary material for: The National Institutes of Health measure of Healing Experience of All Life Stressors (NIH-HEALS): Factor analysis and validation
Source: PLoS One. 2018 Dec 12;13(12):e0207820. doi: 10.1371/journal.pone.0207820 (PMC6291293; doi:10.1371/journal.pone.0207820)
Supplement: S2 File — (DOCX) [file pone.0207820.s002.docx]

**Demographic Questionnaire**

**How old are you?**

**What is your gender?** □Male □Female □Other (describe)__________________________

**What is your race?**

□American Indian/Alaska Native □Asian □Black or African American □Caucasian □Native Hawaiian/Pacific Islander □Mixed/Two or more (describe) __________________________ □Other (describe) __________________________

**Are you Hispanic or Latino?** □Yes □No

**Where were you born?**

**What is your marital status?**

□Single □Married □Divorced/Separated □Widowed □Living with partner □Other (describe)_____________________

**What is the highest level of education you have completed?**

□ Grade school □ High school/GED □ Vocational training

□ Some college/university □ Completed college/university □ Graduate school/advanced degree

□ Other (describe)__________________________

**What is your religion?**

□Christianity □Islam □Hinduism □Buddhism □Judaism □Agnostic □Atheist □Not affiliated □Other (describe) __________________________

**What is your employment status?**  □Full time □Part time □Not employed □Other (describe)_____________________

**If employed, what kind of work do you do?**

**What is your current, most severe illness**?

**When were you diagnosed with your current, most severe illness (as best as you can remember)**?

**How severe is your medical illness?**

□Not severe □Mild □Moderate □Severe □Extremely severe/life limiting

**What other medical diagnosis/diagnoses do you have?—List the most troubling ones.**

**1.**

**2.**

**3.**

**4.**

**Others (please list):**

**Are you experiencing pain?**

□No pain □Some pain □Moderate pain □Severe pain □Extremely severe

**Do you have a current psychiatric diagnosis?** □Yes □No

**If yes, what are you diagnosed with?**

**1.**

**2.**

**3.**

**4.**

**Others (please list):**

**If yes, indicate severity of your psychiatric illness?**

□Not severe □Mild □Moderate □Severe □Extremely severe

**Do you have a history of psychiatric illness?** □Yes □No

**If yes, what were you diagnosed with?**

□Same as current listed above

If others, please list below:

**1.**

**2.**

**3.**

**4.**

**When were you first diagnosed with a psychiatric condition (date diagnosed as best as you can remember)**?

**What losses have you experienced in life? Please write none, if no losses.**

**None.**

**1.**

**2.**

**3.**

**4.**

**5.**

**6.**

**7.**

**Please write the total number of losses:**

**Please rate your current level of stress:**

□No stress □Mild □Moderate □Severe □Extreme

**Please list 3 main sources of stress:**

**1.**

**2.**

**3.**

**Others (please list):**

**What is your current level of social support (friends, family, community, religion/spirituality, other)?**

□No support □Some support □Good support □Excellent support

**Please list the 3 main sources of support:**

**1.**

**2.**

**3.**

**Other** (describe)__________________________

**How do you rate your current overall health status?**

□Poor □Manageable □Satisfactory/Fair □Good □Excellent

**How do you rate your quality of life?**

□Poor □Manageable □Satisfactory/Fair □Good □Excellent
