# Supplementary material for: The National Institutes of Health measure of Healing Experience of All Life Stressors (NIH-HEALS): Factor analysis and validation
Source: PLoS One. 2018 Dec 12;13(12):e0207820. doi: 10.1371/journal.pone.0207820 (PMC6291293; doi:10.1371/journal.pone.0207820)
Supplement: S3 File — (DOCX) [file pone.0207820.s003.docx]

|  | **Strongly Disagree** | **Disagree** | **Neither Agree or Disagree** | **Agree** | **Strongly Agree** |
| --- | --- | --- | --- | --- | --- |
| 1. I am content with my life. | 1 | 2 | 3 | 4 | 5 |
| 1. I have a sense of purpose in my life. | 1 | 2 | 3 | 4 | 5 |
| 1. The connection with a higher power is important to me. | 1 | 2 | 3 | 4 | 5 |
| 1. I gain awareness from self-reflection. | 1 | 2 | 3 | 4 | 5 |
| 1. I enjoy activities that involve both mind/ body such as meditation, prayer, yoga, tai chi, chanting. | 1 | 2 | 3 | 4 | 5 |
| 1. I feel isolated. | 1 | 2 | 3 | 4 | 5 |
| 1. I feel calm even though I am not in control of my situation. | 1 | 2 | 3 | 4 | 5 |
| 1. I accept things that I cannot change. | 1 | 2 | 3 | 4 | 5 |
| 1. Working through thoughts about the possibility of dying brought meaning to my life. | 1 | 2 | 3 | 4 | 5 |
| 1. Difficult circumstances in my life have increased my compassion towards others. | 1 | 2 | 3 | 4 | 5 |
| 1. I want to make the most of my life. | 1 | 2 | 3 | 4 | 5 |
| 1. I survive difficult circumstances because of a higher power. | 1 | 2 | 3 | 4 | 5 |
| 1. My situation strengthened my connection to a higher power. | 1 | 2 | 3 | 4 | 5 |
|  | **Strongly Disagree** | **Disagree** | **Neither Agree or Disagree** | **Agree** | **Strongly Agree** |
| 1. My religious beliefs help me feel calm when faced with difficult circumstances in life. | 1 | 2 | 3 | 4 | 5 |
| 1. My personal religious practice is important to me. | 1 | 2 | 3 | 4 | 5 |
| 1. My participation in a religious community is an important aspect of my life. | 1 | 2 | 3 | 4 | 5 |
| 1. I get support from my religious community. | 1 | 2 | 3 | 4 | 5 |
| 1. My religious beliefs give me hope. | 1 | 2 | 3 | 4 | 5 |
| 1. Doing something I am passionate about gives me purpose during difficult times (e.g. work, hobbies, volunteering, my religious institution, reading groups). | 1 | 2 | 3 | 4 | 5 |
| 1. I find meaning in helping others. | 1 | 2 | 3 | 4 | 5 |
| 1. Connection with my family has become my highest priority. | 1 | 2 | 3 | 4 | 5 |
| 1. Support from my family lifts my spirits, which gives me hope during difficult times in life. | 1 | 2 | 3 | 4 | 5 |
| 1. I am not getting the support I need. | 1 | 2 | 3 | 4 | 5 |
| 1. I am confident that my medical caregivers will respond to my needs. | 1 | 2 | 3 | 4 | 5 |
|  | **Strongly Disagree** | **Disagree** | **Neither Agree or Disagree** | **Agree** | **Strongly Agree** |
| 1. My friends provide the support I need during difficult times. | 1 | 2 | 3 | 4 | 5 |
| 1. I seek more of a connection in my relationships. | 1 | 2 | 3 | 4 | 5 |
| 1. I take more time to be in the moment. | 1 | 2 | 3 | 4 | 5 |
| 1. My experience with multiple losses has made it hard to be hopeful during difficult times (such as death, divorce, competency, physical disability). | 1 | 2 | 3 | 4 | 5 |
| 1. Working through my own grief has brought meaning to my life. | 1 | 2 | 3 | 4 | 5 |
| 1. I have a sense of peace in my life. | 1 | 2 | 3 | 4 | 5 |
| 1. I have an increased sense of gratitude. | 1 | 2 | 3 | 4 | 5 |
| 1. Being surrounded by nature is meaningful. | 1 | 2 | 3 | 4 | 5 |
| 1. Creative arts bring peace to my life. | 1 | 2 | 3 | 4 | 5 |
| 1. Life challenges interfere with activities that are important to me. | 1 | 2 | 3 | 4 | 5 |
| 1. Life challenges raised my desire to be more positive. | 1 | 2 | 3 | 4 | 5 |
