# Supplementary material for: The National Institutes of Health measure of Healing Experience of All Life Stressors (NIH-HEALS): Factor analysis and validation
Source: PLoS One. 2018 Dec 12;13(12):e0207820. doi: 10.1371/journal.pone.0207820 (PMC6291293; doi:10.1371/journal.pone.0207820)
Supplement: S4 File — (DOCX) [file pone.0207820.s004.docx]

Below is a list of statements. By circling **one number per question**, please indicate how much you agree with each statement as it applies to you now since your life changing experience such as physical disability, traumatic events, life-limiting illness, death, divorce, significant losses, or any other life altering experiences. There is no right or wrong answer to these statements. Your response is based on your unique experiences, so it may not reflect responses of others.

|  | **Strongly Disagree** | **Disagree** | **Neither Agree or Disagree** | **Agree** | **Strongly Agree** |
| --- | --- | --- | --- | --- | --- |
| 1. I am content with my life. | 1 | 2 | 3 | 4 | 5 |
| 1. I have a sense of purpose in my life. | 1 | 2 | 3 | 4 | 5 |
| 1. I feel less stressed when I connect with others. | 1 | 2 | 3 | 4 | 5 |
| 1. It is difficult to ask others for help because I do not want to burden them. | 1 | 2 | 3 | 4 | 5 |
| 1. The connection with a higher power is important to me. | 1 | 2 | 3 | 4 | 5 |
| 1. I gain awareness from self-reflection. | 1 | 2 | 3 | 4 | 5 |
| 1. I enjoy activities that involve both mind/ body such as meditation, prayer, yoga, tai chi, chanting. | 1 | 2 | 3 | 4 | 5 |
| 1. I feel isolated. | 1 | 2 | 3 | 4 | 5 |
|  | **Strongly Disagree** | **Disagree** | **Neither Agree or Disagree** | **Agree** | **Strongly Agree** |
| 1. I feel calm even though I am not in control of my situation. | 1 | 2 | 3 | 4 | 5 |
| 1. I accept things that I cannot change. | 1 | 2 | 3 | 4 | 5 |
| 1. Working through thoughts about the possibility of dying brought meaning to my life. | 1 | 2 | 3 | 4 | 5 |
| 1. Difficult circumstances in my life have increased my compassion towards others. | 1 | 2 | 3 | 4 | 5 |
| 1. I have a greater appreciation for my life. | 1 | 2 | 3 | 4 | 5 |
| 1. I want to make the most of my life. | 1 | 2 | 3 | 4 | 5 |
| 1. I no longer focus on “the little things.” | 1 | 2 | 3 | 4 | 5 |
| 1. I survive difficult circumstances because of a higher power. | 1 | 2 | 3 | 4 | 5 |
| 1. My situation strengthened my connection to a higher power. | 1 | 2 | 3 | 4 | 5 |
| 1. My religious beliefs help me feel calm when faced with difficult circumstances in life. | 1 | 2 | 3 | 4 | 5 |
| 1. My personal religious practice is important to me. | 1 | 2 | 3 | 4 | 5 |
|  | **Strongly Disagree** | **Disagree** | **Neither Agree or Disagree** | **Agree** | **Strongly Agree** |
| 1. My values shape the way I live my life. | 1 | 2 | 3 | 4 | 5 |
| 1. My participation in a religious community is an important aspect of my life. | 1 | 2 | 3 | 4 | 5 |
| 1. I get support from my religious community. | 1 | 2 | 3 | 4 | 5 |
| 1. My religious beliefs give me hope. | 1 | 2 | 3 | 4 | 5 |
| 1. Doing something, I am passionate about (such as work, hobbies, volunteering, my religious institution, reading groups) gives me purpose during difficult times. | 1 | 2 | 3 | 4 | 5 |
| 1. I find meaning in helping others. | 1 | 2 | 3 | 4 | 5 |
| 1. Relationships with my friends are more meaningful since my challenging situation began. | 1 | 2 | 3 | 4 | 5 |
| 1. Connection with my family has become my highest priority. | 1 | 2 | 3 | 4 | 5 |
| 1. Support from my family lifts my spirits, which gives me hope during difficult times in life. | 1 | 2 | 3 | 4 | 5 |
|  | **Strongly Disagree** | **Disagree** | **Neither Agree or Disagree** | **Agree** | **Strongly Agree** |
| 1. I am not getting the support I need. | 1 | 2 | 3 | 4 | 5 |
| 1. I am confident that my medical caregivers will respond to my needs. | 1 | 2 | 3 | 4 | 5 |
| 1. My friends provide the support I need during difficult times. | 1 | 2 | 3 | 4 | 5 |
| 1. I seek more of a connection in my relationships. | 1 | 2 | 3 | 4 | 5 |
| 1. I take more time to be in the moment. | 1 | 2 | 3 | 4 | 5 |
| 1. My experience with multiple losses (such as death, divorce, competency, physical disability) has made it hard to be hopeful during difficult times. | 1 | 2 | 3 | 4 | 5 |
| 1. Working through my own grief has brought meaning to my life. | 1 | 2 | 3 | 4 | 5 |
| 1. I have a sense of peace in my life. | 1 | 2 | 3 | 4 | 5 |
| 1. I have an increased sense of gratitude. | 1 | 2 | 3 | 4 | 5 |
|  | **Strongly Disagree** | **Disagree** | **Neither Agree or Disagree** | **Agree** | **Strongly Agree** |
| 1. Relationship with my family is more meaningful. | 1 | 2 | 3 | 4 | 5 |
| 1. Being surrounded by nature is meaningful. | 1 | 2 | 3 | 4 | 5 |
| 1. Creative arts bring peace to my life. | 1 | 2 | 3 | 4 | 5 |
| 1. Life challenges interfere with activities that are important to me. | 1 | 2 | 3 | 4 | 5 |
| 1. Life challenges raised my desire to be more positive. | 1 | 2 | 3 | 4 | 5 |
