# Supplementary material for: The National Institutes of Health measure of Healing Experience of All Life Stressors (NIH-HEALS): Factor analysis and validation
Source: PLoS One. 2018 Dec 12;13(12):e0207820. doi: 10.1371/journal.pone.0207820 (PMC6291293; doi:10.1371/journal.pone.0207820)
Supplement: S5 File — (DOCX) [file pone.0207820.s005.docx]

**Please circle or mark one number per line to indicate your response as it applies to the past 7 days.**

**Not at A little Some- Quite Very**

**all bit what a bit much**

| Sp1  Sp2  Sp3  Sp4  Sp5  Sp6  Sp7  Sp8  Sp9  Sp10  Sp11  Sp12 |
| --- |

I feel peaceful...................................................................... 0 1 2 3 4

I have a reason for living..................................................... 0 1 2 3 4

My life has been productive................................................ 0 1 2 3 4

I have trouble feeling peace of mind................................... 0 1 2 3 4

I feel a sense of purpose in my life ..................................... 0 1 2 3 4

I am able to reach down deep into myself for comfort ....... 0 1 2 3 4

I feel a sense of harmony within myself ............................. 0 1 2 3 4

My life lacks meaning and purpose..................................... 0 1 2 3 4

I find comfort in my faith or spiritual beliefs...................... 0 1 2 3 4

I find strength in my faith or spiritual beliefs ..................... 0 1 2 3 4

My illness has strengthened my faith or spiritual beliefs.... 0 1 2 3 4

I know that whatever happens with my illness, things

will be okay......................................................................... 0 1 2 3 4
