# Supplementary material for: The National Institutes of Health measure of Healing Experience of All Life Stressors (NIH-HEALS): Factor analysis and validation
Source: PLoS One. 2018 Dec 12;13(12):e0207820. doi: 10.1371/journal.pone.0207820 (PMC6291293; doi:10.1371/journal.pone.0207820)
Supplement: S6 File — (DOCX) [file pone.0207820.s006.docx]

| **Mark the box in the column that most closely describes how often the statement describes the way you are.** **If you are uncertain how to answer, mark your best guess*―*please respond to each statement.** | | | | | |
| --- | --- | --- | --- | --- | --- |
| ***This statement describes the way I am:*** | **Very Rarely** | **Some-**  **times** | **About**  **Half the Time** | **More**  **Often**  **Than Not** | **Most of**  **the Time** |
| 1. When my future is uncertain, I have a basic sense of trust that things will turn out OK. | **○** | **○** | **○** | **○** | **○** |
| 2. When someone hurts me, I have a hard time forgetting about it. | **○** | **○** | **○** | **○** | **○** |
| 3. I have trouble forgetting about my mistakes. | **○** | **○** | **○** | **○** | **○** |
| 4. I feel that others control my life. | **○** | **○** | **○** | **○** | **○** |
| 5. I know someone who will take the time to understand all my pains, sorrows and joys. | **○** | **○** | **○** | **○** | **○** |
| 6. I have a purpose or mission for my life. | **○** | **○** | **○** | **○** | **○** |
| 7. Talking about my troubles with someone I trust helps me to understand myself and my life in a new way. | **○** | **○** | **○** | **○** | **○** |
| 8. I like to laugh and play. | **○** | **○** | **○** | **○** | **○** |
| 9. Someone supports me when I try to change my behavior. | **○** | **○** | **○** | **○** | **○** |
| 10. I know how to ask for help when I need it. | **○** | **○** | **○** | **○** | **○** |
| 11. I find myself repeatedly doing things that aren't good for me. | **○** | **○** | **○** | **○** | **○** |
| 12. When I think about certain episodes from earlier in my life I still get upset and angry. | **○** | **○** | **○** | **○** | **○** |
| 13. I have trouble relaxing. | **○** | **○** | **○** | **○** | **○** |
| 14. I feel incomplete as a person. | **○** | **○** | **○** | **○** | **○** |
| 15. I've learned how to take care of myself emotionally. | **○** | **○** | **○** | **○** | **○** |
| 16. I believe that life is often not fair to me. | **○** | **○** | **○** | **○** | **○** |
| 17. I feel like people often take advantage of me. | **○** | **○** | **○** | **○** | **○** |
| 18. I have someone that I can tell my deepest darkest secrets and still feel safe. | **○** | **○** | **○** | **○** | **○** |
| SIS v 2.1. © James Meza 2007. All rights reserved. May be used with permission for all research funded by nonprofit sources by citing: Meza JP, Fahoome GF. The development of an instrument for measuring healing. *Ann Fam Med.* 2008;6(4):355-360 . All others require written permission. | | | | | |
